# Safety of COVID-19 Vaccines among People with History of Allergy: A European Active Surveillance Study

**DOI:** 10.3390/vaccines12091059

**Published:** 2024-09-17

**Authors:** Nicoletta Luxi, Francesco Ciccimarra, Chiara Bellitto, Monika Raethke, Florence van Hunsel, Thomas Lieber, Erik Mulder, Luca L’Abbate, Francisco Batel Marques, Fabiana Furci, Andreea Farcas, Janneke Giele-Eshuis, Kathryn Morton, Simona Sonderlichová, Nicolas H. Thurin, Felipe Villalobos, Fabio Riefolo, Miriam C. Sturkenboom, Gianluca Trifirò

**Affiliations:** 1 Department of Medicine, University of Verona, 37134 Verona, Italy; 2 Department of Diagnostics and Public Health, University of Verona, 37134 Verona, Italy; 3 Netherlands Pharmacovigilance Centre Lareb, Goudsbloemvallei 7, 5237 MH ’s-Hertogenbosch, The Netherlands; 4 Department of PharmacoTherapy, Epidemiology & Economics, Groningen Research Institute of Pharmacy (GRIP), University of Groningen, 9712 Groningen, The Netherlands; 5 Laboratory of Social Pharmacy and Public Health, School of Pharmacy, University of Coimbra, 3000-548 Coimbra, Portugal; 6 Provincial Healthcare Unit, Section of Allergy, 89900 Vibo Valentia, Italy; 7 Pharmacovigilance Research Center, Iuliu Hatieganu University of Medicine and Pharmacy, 400347 Cluj-Napoca, Romania; 8 Department of Data Science and Biostatistics, Julius Global Health, University Medical Centre Utrecht, 3584 Utrecht, The Netherlands; 9 Drug Safety Research Unit, Southampton SO31 1AA, UK; 10 University of Portsmouth, Portsmouth PO1 2UP, UK; 11 Faculty of Medicine, SLOVACRIN, Pavol Jozef Šafárik University in Košice, 040 01 Košice, Slovakia; simona.sonderlichova@upjs.sk; 12 University of Bordeaux, INSERM CIC-P 1401, Bordeaux PharmacoEpi, 146 rue Léo Saignat, 33076 Bordeaux, France; 13 Fundació Institut Universitari per a la Recerca a l’Atenció Primària de Salut Jordi Gol i Gurina (IDIAPJGol), 08007 Barcelona, Spain; fvillalobos@idiapjgol.info; 14 Teamit Institute, Partnerships, Barcelona Health Hub, 08025 Barcelona, Spain

**Keywords:** COVID-19 vaccines, adverse reactions, allergy history, safety profile, anaphylaxis

## Abstract

**Background:** Conventional vaccines rarely cause severe allergic reactions. However, the rapid development and approval of COVID-19 vaccines left limited initial data on their adverse reactions, particularly in individuals with a history of allergy. The aim of this study was to assess and compare the safety profile of different doses and brands of COVID-19 vaccines in subjects with a history of allergy vs. those without a history of allergy. **Methods:** From February 2021 to February 2023, a web-based prospective study gathered vaccinee-reported outcomes using electronic questionnaires across eleven European countries. Baseline and up to six follow-up questionnaires captured data on vaccinee demographics, as well as both solicited and unsolicited adverse reactions. **Results:** Overall, 3476 vaccinees with a history of allergy were matched with 13,872 vaccinees from the general population at the first vaccination cycle and were included in the analysis. A total of 825 vaccinees with a history of allergy who had received a booster dose, matched to 3297 vaccinees from the general population, were included in the analysis. Higher rates of ADRs occurred after the first vaccination cycle compared to after the booster dose (64–91% vs. 56–79%). However, most reported ADRs were solicited and not serious, and no case of anaphylaxis was reported. Women and vaccinees with a history of allergy reported ADRs more frequently than men and the matched controls, respectively. Compared to other COVID-19 vaccines, a higher proportion of vaccinees experiencing at least one ADR following their first vaccination cycle was observed with Comirnaty and Vaxzevria. Statistically significant differences were observed among the study cohorts for median TTO after the second dose, and for median TTR following the first vaccination cycle and booster dose (*p* < 0.001). **Conclusions:** Typically, any drug or vaccine use carries a risk of severe allergic reactions, yet the benefits of vaccination generally outweigh these potential risks, as shown with the COVID-19 vaccines.

## 1. Background

Conventional vaccines are known to occasionally cause allergic reactions that are generally mild (e.g., hypersensitivity reactions) and mostly related to the vaccine components and, only rarely, severe, such as anaphylaxis [1,2,3,4,5]. In particular, a rate of 1.4 severe allergic reactions per million doses of conventional vaccines has been estimated. Based on innovative vaccine technologies, mRNA COVID-19 vaccines were developed and conditionally approved for marketing by regulatory agencies at an unprecedented pace. Hence, limited information regarding short- and long-term adverse reactions (ADRs), including allergic reactions, at the time that these vaccines were marketed were available, thus warranting intensive post-marketing safety surveillance. While the vaccines demonstrated efficacy in preventing severe illness and reducing the spread of the virus, additional information about the risks associated with the COVID-19 vaccines in special populations is required. In particular, although serious adverse allergic reaction occurred rarely, concerns regarding the safety of COVID-19 vaccination in people with a prior history of allergies have been raised. Having allergies to drugs, foods, insect venoms, or inhalant allergens (such as house dust mites, pollens, animal dander, and molds) is generally not a contraindication for vaccines. The mechanisms underlying these reactions are not fully understood. However, certain excipients like polyethylene glycol (PEG), polysorbates, and tromethamine/trometamol have been identified as potential causes of systemic allergic reactions [2]. Regulatory agencies and guidelines recommended COVID-19 vaccination to be avoided only in two specific situations: allergy to one of the components of the vaccine, supported by appropriate allergy tests, and a history of a severe allergic reaction to the first dose. In addition, vaccination should be administered under close medical supervision, and appropriate medical assistance should be available during the vaccination process. This ensures that any potential adverse reactions to vaccines can be promptly addressed [6]. In pivotal clinical trials of COVID-19 vaccines, only an extremely low number of cases of anaphylaxis in both the vaccine and placebo groups were reported. This was expected considering that people with history of severe hypersensitivity reactions were excluded from pivotal clinical trials. Only one phase II trial on COVID-19 vaccines included people with a history of allergy or mast cell disorder to assess systemic allergic reactions to COVID-19 vaccines in such a population vs. those without severe allergies or mast cell disorders [7]. The trial concluded that there were no statistically significant differences in the risk of developing systemic allergic reactions between those with a history of allergy and the control group. However, at the beginning of COVID-19 vaccination campaign in December 2020, serious adverse allergic reactions following vaccine administration were reported [8]. An analysis from the Vaccine Adverse Event Reporting System (VAERS) database found that, during a 6-month period, there were more than 14,000 reports of allergic reactions following a COVID-19 vaccination [9]. The results showed that people with a history of allergies, asthma, and anaphylaxis were more likely to experience allergic reactions compared to those with no such medical history. In line with spontaneous reporting, observational studies found that COVID-19 vaccine allergic reactions may be more prevalent among patients with a history of allergy than the general population [10,11,12]. However, most of these observational studies were carried out in individual centers, focused on evaluating the safety of only one or, at most, two vaccine brands, and had a limited follow-up period. Accordingly, a Turkish active surveillance study including people with a history of allergy reported that a known history of allergy increased the risk of having an allergic reaction by approximately six-fold following vaccination [13]. However, in this study, information on the COVID-19 vaccine dose, other previous vaccinations, and a history of allergic reaction following previous COVID-19 vaccine doses was not assessed.

To date, no observational studies evaluating the comparative safety of all European Medicines Agency (EMA)-authorized COVID-19 vaccines across different brands and doses specifically in individuals with a history of allergy have been published.

The EMA funded the “COVID Vaccine Monitor” (CVM), a comprehensive multi-country project (EUPAS42504). The CVM is a large-scale cohort event monitoring study that aims to collect self-reported outcomes from vaccinees regarding the safety of all EMA-authorized COVID-19 vaccines from the general population and special cohorts [14,15,16], including individuals with a history of allergy, from eleven European countries. In the context of CVM, the objective of this study was to evaluate the safety of different doses and brands of COVID-19 vaccines in subjects with a history of allergy, matched in a 1:4 ratio to those without prior allergies.

## 2. Methods 

### 2.1. Study Design and Study Population

This was a prospective observational cohort study based on patient-reported outcomes via web-based questionnaires in 11 European countries (Supplementary Table S1) from February 2021 to February 2023. People who reported a history of allergy at the time of study enrollment, who received a first/booster dose of any EMA-authorized COVID-19 vaccine at the time of the study period (Comirnaty, Jcovden, Novavax, Spikevax, and Vaxzevria), and who registered within 48 h after the vaccine administration and provided informed consent were recruited between February 2021 and November 2022. No age restriction was applied. In the case of vaccinees aged under 18 years old, parents or legal representatives could participate in the study as proxies. The same inclusion criteria were applied to select vaccinees from the general population with no history of allergy and participating in CVM as a control group.

### 2.2. Data Collection and Processing

Two dedicated and standardized web-based applications, namely the Lareb-managed Intensive Monitoring (LIM) and ResearchOnline (RO) applications, were created for the data collection. These web apps were designed specifically to capture the outcomes reported by vaccinees and were translated into several languages. Vaccinees were invited to participate in the study through appropriate information materials (e.g., flyers and posters) distributed at vaccination centers and through different online channels. A baseline questionnaire collecting information on the vaccinees’ characteristics, including demographics, medical history, concomitant drug use, and administered COVID-19 vaccine dose and brand, was sent shortly after registration to the study. In addition, for people with a history of allergy, questions on the type of allergy (i.e., hay fever, dust mite allergy, animal allergy, food allergy, insect bite allergy, drug allergy, and/or other allergies), previous allergic reactions after receiving any vaccine, previous allergic reactions (e.g., anaphylactic shock) that required emergency treatment or attendances and emergency admission, and any pre-medication (e.g., antihistamines or corticosteroids) before COVID-19 vaccination to prevent vaccine-related allergy, were specifically asked.

Then, for the monitoring of short-/medium-/long-term suspected ADRs related to the COVID-19 vaccine administration, six follow-up questionnaires (over a 6-month period) and five follow-up questionnaires (over a 3-month period) after the first and booster doses of COVID-19 vaccines, respectively, were sent at different time points. Information on solicited (close-ended questions) local (injection site-related reactions) and systemic (arthralgia, chills, fatigue, headache, malaise, myalgia, nausea, and fever) ADRs was collected. Unsolicited (open-ended questions) ADRs were also collected, as well as adverse events of special interest (AESIs). Moreover, information about the timing, duration, and burden of ADRs was collected as well. The solicited ADRs were automatically coded using the Medical Dictionary for Regulatory Activities (MedDRA) [17], while unsolicited ADRs were manually assessed. Seriousness was classified by qualified personnel, including pharmacovigilance-trained personnel and study investigators from each participating institution following the Council for International Organizations of Medical Sciences (CIOMS) seriousness criteria [18]. In addition, several countries reported collected ADRs to EudraVigilance in accordance with their national regulations. Further details on the assessment of the information reported in the questionnaires and the data processing have been provided in a previous publication [19].

### 2.3. Data Analysis

Given the utilization of multiple primary data collection tools with closely aligned structures, a common data model (CDM) was developed to facilitate data harmonization. The implementation of a CDM allows for the creation of simplified person-centric record-based tables, enhancing accessibility for analyses. Additional information has been reported elsewhere [19].

To compare the baseline characteristics and rate of ADRs of vaccinees with a history of allergy with those with no history of allergy in the general population, the propensity score (PS) methodology was applied. Based on PS values, controls were 1:4 matched to vaccinees with a history of allergy by gender, age, and vaccine brand and dose, using the nearest neighbor matching procedure. Overall, the number of recruited vaccinees and baseline and follow-up questionnaires completed was reported. Analyses were restricted to vaccinees enrolled in the study, focusing specifically on those who completed the baseline questionnaire and, at a minimum, the first follow-up questionnaire (referred to as Q1). Vaccinees who reported receiving Novavax or Vaxzevria vaccines were excluded from the analyses due to the limited sample size, as well as those who reported an unknown COVID-19 vaccine.

The incidences of patient-reported local and systemic ADRs, as well as serious and unsolicited ADRs, following the first, the second (if applicable), and the booster doses of different COVID-19 vaccines, stratified by gender and vaccine dose, were measured as the proportion of the number of ADRs over the total number of subjects in the cohorts. Scatterplots of the frequency of vaccinee-reported ADRs, as a whole and as local and systemic solicited ADRs, stratified by gender and vaccine dose, were generated. Among the unsolicited ADRs, the rate of allergy-related ADRs at preferred term (PT) level was also calculated. The median time to onset (TTO) and the median time to recovery (TTR) of reported ADRs, along with their interquartile range (IQR) in hours, were assessed and displayed using a combination of violin plots and boxplots. Only vaccinees who reported both TTO and TTR for each reported ADR were considered. Categorical variables were presented as absolute frequencies and percentages, while continuous variables were expressed as the median (with interquartile ranges) where appropriate. A comparison of categorical variables was conducted using either the χ^2^ test or Fisher’s exact test, as appropriate. Statistical significance was indicated by a *p*-value < 0.05. Analyses were executed using R statistical software (version 4.3.1). The ggplot2 R package was utilized for generating the heatmaps, violin plots, and boxplots.

## 3. Results

### 3.1. Baseline Characteristics of Recruited Vaccinees

**Figure 1:**
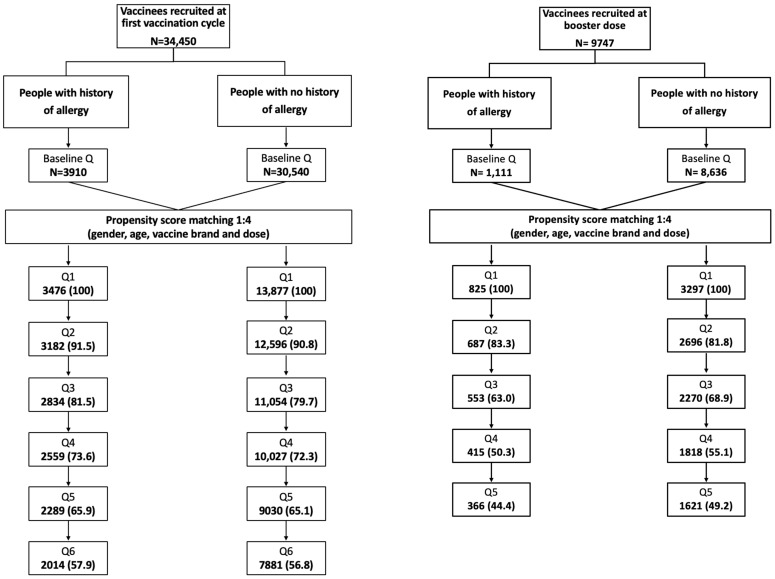
Flowchart of vaccinees recruited in the study who completed baseline and follow-up questionnaires.

A total of 44,197 subjects registered for the study and completed the baseline questionnaire (Figure 1); of these, 77.9% registered after receiving their first vaccination and 22.1% after receiving a booster dose of a COVID-19 vaccine. However, only vaccinees who completed at least Q1 were included in the analyses, resulting in a total of 21,475 subjects. Of those included at the first vaccination cycle (N = 17,353, 80.8%), 3476 reported a history of allergy, while 13,877 belonged to the matched general population cohort. Regarding vaccinees included at the booster dose (N = 4122, 19.2%), 825 with a history of allergy were matched to 3297 without a history of allergy. On average, 57% and 47% of vaccinees completed all follow-up questionnaires, respectively, for the first vaccination cycle and booster dose. The demographic and clinical characteristics of the included vaccinees are shown in Table 1. Overall, the median age category was between 34 and 59 years and more than 70% of the included vaccinees were female. At the first vaccination cycle, the most frequently reported COVID-19 vaccines were Comirnaty (43%) and Vaxzevria (32%), followed by Spikevax (15%) and Jcovden (10%). Regarding the booster dose, Comirnaty (52%) and Spikevax (48%) were the most frequently reported (Table 2).

### 3.2. Analysis of Frequency of Reported ADRs

**Figure 2:**
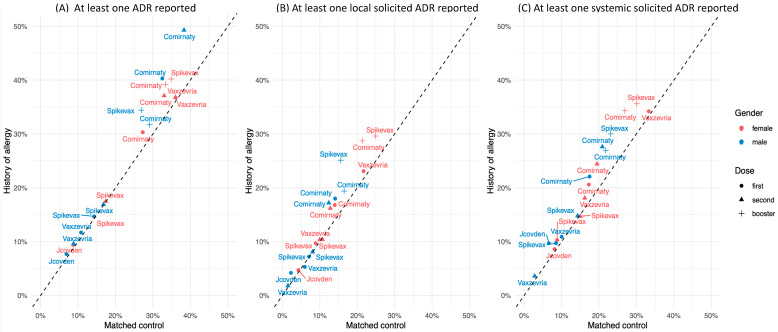
Proportions of female and male participants who reported at least one ADR (**A**), at least one local solicited ADR (**B**) and at least one systemic solicited ADR (**C**) after receiving a first, second or booster dose of different COVID-19 vaccines, among people with a history of allergy and matched controls.

Overall, a higher rate of ADRs was observed after the first vaccination cycle, including the first and second doses, compared with the booster dose (ranging from 64 to 91% vs. 56 to 79%), with a higher frequency among women than men and among vaccinees with a history of allergy vs. the matched controls (Figure 2A, corresponding to Supplementary Table S2). The proportion of vaccinees reporting at least one ADR following Comirnaty and Vaxzevria vaccination at the first vaccination cycle was higher compared to the other COVID-19 vaccines. A higher ADR rate was observed among female allergic vaccinees following the second or booster dose of Comirnaty compared to the first dose (37% vs. 39% vs. 30%, respectively); among male allergic vaccinees, higher ADR rates after the second dose (49%) and lower rates after the booster dose (32%) were observed compared to the first dose (40%). Among Vaxzevria recipients, the ADR rate was higher in female vaccinees than males (37% vs. 12%). Regarding the booster dose, slightly higher rates following Spikevax compared to Comirnaty were observed. The same trend, with slightly lower rates, was observed among the matched controls. Most of the reported ADRs were solicited, with a higher frequency of systemic than local ADRs (Figure 2B,C, corresponding to Supplementary Tables S3 and S4). Solicited ADRs, both local and systemic, were mostly related to the injections site pain, fatigue, headache, malaise, and myalgia (Supplementary Table S5). Local solicited ADRs (Figure 2B) were most frequently reported following a first dose of Comirnaty and Vaxzevria and more frequently by female vaccinees compared to male vaccinees, and by those with a history of allergy compared to the matched controls. The same trend was observed for the second and booster doses, with slightly lower rates following second doses compared to first doses. Regarding systemic ADRs (Figure 2C), they were reported most frequently after the first dose of Comirnaty and Vaxzevria, with a higher rate among females than males in both the vaccinees with a history of allergy and matched controls. The same trend was observed following the second and booster doses, with higher frequencies following the second dose of Comirnaty and Spikevax than after the first dose. The same trend was also observed for serious ADRs (Table 3), which were more frequently reported among females than males in both vaccinees with a history of allergy and the matched controls. However, the frequency of the reported serious ADRs was very low: overall, 0.2% of allergic vs. 0.1% of non-allergic vaccinees, and 0.3% vs. 0.1% and 0.3% vs. 0.2% in the two cohorts reported at least one serious ADR following the first and second or booster dose, respectively. More than 50% of the vaccinees reported at least one unsolicited ADR after a first dose of Jcovden (60% vs. 50%) and Vaxzevria (66% vs. 59%), both in those with a history of allergy and the matched controls (Table 4). Lower rates of unsolicited ADRs were observed following the second dose of Vaxzevria compared to the first dose in both the people with a history of allergy and matched controls (21% vs. 66% and 15% vs. 59%, respectively); higher rates were reported following the second dose of Spikevax (Table 5) compared to the first dose (54% and 50% vs. 32% and 25% in allergic people and matched controls, respectively). Considering the booster dose, lower frequencies of unsolicited ADRs were observed following Comirnaty and Spikevax (Table 6). Among the unsolicited allergy-related ADRs, dyspnea was the most frequently reported following the first and second doses of different COVID-19 vaccine brands and doses. In particular, it was significantly higher in the allergic vaccinees than matched controls following the first dose of the Jcovden and Vaxzevria vaccines, and the second dose of Comirnaty and Vaxzevria. Regarding the booster dose, cough was the most frequently reported reaction following the Comirnaty vaccine among the allergic vaccinees, with no significant differences compared to the matched controls; eczema and pruritus were the most frequently reported ADR following Spikevax vaccination, and their frequencies were higher in the allergic people than in the matched controls. Only one anaphylactic reaction was reported among the people with a history of allergy following the administration of the first dose of Jcovden. In addition, one anaphylactoid reaction was also reported following a first and a second dose of Comirnaty. No anaphylactic reactions were reported among the matched controls, nor after the booster dose.

### 3.3. Analysis of Time-to-Onset and Time-to-Recovery of the Reported ADRs

**Figure 3:**
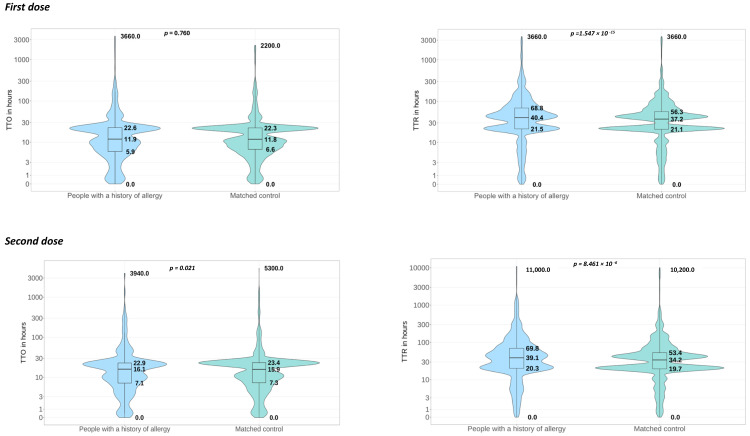

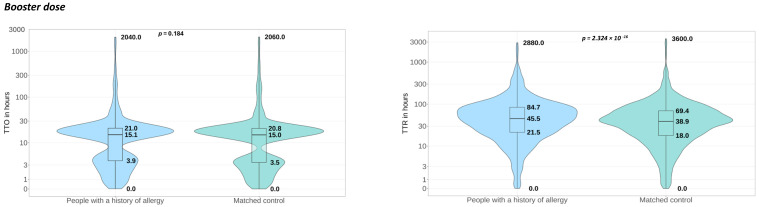
Combination of violin plots and boxplots depicting the median time to onset and median time to recovery, measured in hours, of ADRs reported by vaccinees after receiving first, second, and booster doses, among people with a history of allergy and matched controls. Abbreviations: TTO = time to onset; TTR = time to recovery.

The TTO and TTR of ADRs across the different doses for vaccinees who reported both TTO and TTR for each reported ADR are shown in Figure 3. The median TTO for all reported ADRs following the first dose was 11.9 (IQR: 5.9–22.6) hours among the vaccinees with a history of allergy and 11.8 (IQR: 6.6–22.3) hours among the matched controls, with no statistically significant difference between the two groups (*p*-value = 0.760). Compared to the first dose, a slightly longer TTO was reported for the second dose with statistically significant differences between the groups: 16.1 (IQR: 7.1–22.9) and 15.9 (IQR: 7.3–23.4), in people with a history of allergy and the matched controls, respectively. The median TTR following the first dose was significantly higher among the people with a history of allergy compared to the matched controls (40.4; IQR: 21.5–68.8 vs. 37.2; IQR: 21.5–56.3; *p*-value = 1.547 × 10^−15^). After the second dose, the overall TTR was slightly lower compared to the first dose in both the people with a history of allergy and the matched controls. However, a significantly higher TTR was observed in the people with a history of allergy compared to the matched controls (39.1; IQR: 20.3–69.8 vs. 34.2; IQR: 19.7–53.4; *p*-value = 8.461 × 10^−6^). Regarding the booster dose, a similar TTO was observed in the people with a history of allergy and the matched controls (15.1; IQR: 3.9–21.0 vs. 15.0; IQR: 3.5–20.8; *p*-value = 0.184), whilst the TTR was significantly higher in the people with a history of allergy compared to the matched controls (45.5; IQR: 21.5–84.7 vs. 38.9; IQR: 18.0–69.4; *p*-value = 2.324 × 10^−16^).

## 4. Discussion

To the best of our knowledge, this is the first cohort event monitoring that provided a comprehensive, comparative assessment of the safety of COVID-19 vaccines across different vaccine doses, in people with a history of allergy compared to a matched cohort from the general population and in different European countries, within a descriptive study framework.

In line with what was reported in the literature [20] and pivotal trials of COVID-19 vaccines in the general population [21,22,23,24], the most frequently reported ADRs in both cohorts were solicited ADRs, including injection site pain, headache, myalgia, and malaise, among the different doses. However, a higher rate of ADRs after the first vaccination cycle compared to the booster dose was observed. In addition, a statistically higher percentage of ADRs was reported among subjects with a history of allergy compared with those in the matched cohort.

Overall, female vaccinees reported ADRs more frequently than males (≃74% vs. 26% at the first vaccination cycle and ≃72% vs. 28% at the booster dose), which is consistent with many observational studies of reported/self-reported allergic reactions to mRNA COVID-19 vaccines that attribute this increased incidence to gender-specific factors, such as hormonal and genetic differences, as well as a history of allergic disease [11,25,26]. Furthermore, the greater proportion of allergic reactions in females may be partly related to the fact that females are more likely to report ADRs than males [27].

Similarly to previous studies [28,29], the majority of the documented ADRs were mild and of short duration. In our study, the TTO of the reported ADRs had a median of 12 h for the first dose and was slightly longer for the second dose, and the TTR generally occurred within a few days.

Although several mild, local, and systemic ADRs have been frequently reported following COVID-19 vaccination, the occurrence of serious ADRs was rare and the percentage of immediate severe allergic reactions was very low. It has indeed been reported that overall, the incidence of serious ADRs, including allergic reactions, after the administration of COVID-19 vaccines remains low, albeit slightly higher than traditional vaccines [1]. Immediate allergic reactions typically occur within 4 h after vaccine administration, with the occurrence of clinical manifestations affecting various organs/systems and related to the degranulation of mast cells and basophils [30,31,32]. In particular, the most severe immediate vaccine-associated reaction is anaphylaxis, with an onset typically within one hour after vaccine administration. As reported in the study by Soria et al., the severity and occurrence of a reaction within one hour after vaccine administration are both key factors in risk stratification of an allergic reaction to a COVID-19 mRNA vaccine [33]. During the early stages of the vaccination campaign, several cases of anaphylaxis were reported after the administration of mRNA COVID-19 vaccines. However, anaphylaxis reactions can also occur after the administration of viral vector COVID-19 vaccines [34]. An analysis of 283 reports of suspected ADRs to mRNA COVID-19 vaccines from the VAERS found 31 cases of anaphylaxis resulting in 5.2 anaphylactic cases per million vaccine doses [35]. A registry-based study conducted using a specific COVID-19 vaccine allergy case registry showed that 11% of vaccinees receiving an mRNA COVID-19 vaccine experienced anaphylaxis [36]. Lower rates of anaphylaxis after the administration of an mRNA COVID-19 vaccine were reported in an observational study based on electronic health records of 64,900 vaccinees with 0.03% identified cases of anaphylaxis [37]. Conversely, in our study, no case of anaphylaxis was reported following Comirnaty vaccination nor after Spikevax vaccination; only one case was reported following Jcovden administration. Furthermore, it was observed that for individuals who experienced an immediate allergic reaction after a first dose of a COVID-19 vaccine, the likelihood of a recurrence of another allergic reaction, including severe reactions like anaphylaxis, following the second dose was low [38,39].

In addition to immediate allergic reactions such as anaphylaxis, delayed adverse reactions, such as delayed cutaneous reactions (e.g., morbilliform rash and urticaria), were also reported in previous studies [40,41,42,43,44,45]. These reactions can occur at about 8 days post-vaccination at the injection site, with erythema, induration, and tenderness [46]. Other allergic-related ADRs were also reported in our study, including cutaneous reactions such as pruritus, rashes, eczema, and urticaria, but with a very low frequency (<1.5%).

One of the key strengths of this study lies in its inclusion of patient-level data from eleven European countries, which were gathered and analyzed using a CDM. Furthermore, due to the flexibility of the LIM and RO web applications, it was possible to integrate and adapt them with the new information made available during the study, facilitating the direct and timely updating of questionnaires. The large sample of vaccinees included in the study allowed for an in-depth analysis of vaccine safety. In addition, by including vaccinees from the general population, it was possible to compare safety outcomes between vaccinees with and without a history of allergy. Moreover, it is worth mentioning the follow-up period among the strengths. Previously published studies investigating the safety of the COVID-19 vaccine in people with a history of allergy were indeed characterized by a limited follow-up period [10,11,12]. Finally, due to the study design, having a denominator available enabled the evaluation of the frequency of particular ADRs. However, some limitations must also be taken into account. Using patient-reported outcomes enables the gathering of safety data that might not be recorded in medical records, which is a crucial aspect, particularly for individuals experiencing short-term and non-serious ADRs post-vaccination who might not seek medical consultation. Participants could enroll in the study within 48 h following vaccination, potentially introducing a selection bias, as those experiencing ADRs shortly after vaccination might be more inclined to register. Moreover, participants experiencing severe ADRs leading to hospitalization might have been unable to complete the questionnaires, potentially resulting in an underestimation of the serious ADR frequency. With a six-month follow-up period, it is improbable that all participants will complete every questionnaire unless highly motivated, leading to a loss to follow-up. Notably, individuals experiencing ADRs may be more inclined to fill out follow-up questionnaires than those without ADRs, introducing a selective loss to follow-up. Similarly, individuals with underlying health conditions may be more likely to report ADRs than those without such conditions. Since self-reporting outcomes may be characterized by recall bias, people with a history of allergy, including those who have experienced a previous allergic reaction following vaccination, may be more likely to report. In addition, there might also have been a bias whereby the symptoms were perceived as an allergic reaction but they might not have been severe or allergic reactions. Lastly, as the data collection relied on electronic tools, some questionnaires might have been lost due to technical disruptions or delivery malfunctions or ended up in spam folders.

## 5. Conclusions

The findings from this study highlighted an overall favorable safety profile of COVID-19 vaccines in people with a history of allergy. The Comirnaty vaccine generally appears to have a more favorable safety profile compared to the Vaxzevria vaccine, showing lower rates of ADRs, particularly among female vaccinees. The Spikevax vaccine showed slightly higher ADR rates compared to the Comirnaty vaccine following the booster dose, but overall, Comirnaty had a more favorable safety profile across different doses and genders.

However, individual responses can vary and careful monitoring is recommended regardless of the type of vaccine used.

In general, the use of any drug or vaccine may carry the risk of severe allergic reactions, although the benefits of vaccination typically outweigh these potential risks, as demonstrated with the COVID-19 vaccines.

## Figures and Tables

**Table 1:** Demographic and clinical characteristics of vaccinees recruited at the first vaccination cycle or booster doses, who completed at least one follow-up questionnaire.

	First Vaccination Cycle ^a^			Booster Dose ^b^		
	People with a History of Allergy N = 3476 *	Matched Controls N = 13,877 **	*p* Value	People with a History of Allergy N = 825	Matched Controls N = 3297	*p* Value
**Gender, n (%)**						
female	2564 (73.8)	10,231 (73.7)	Matching factor	596 (72.2)	2381 (72.2)	Matching factor
male	912 (26.2)	3646 (26.3)		229 (27.8)	916 (27.8)	
**Median, years (IQR)**	48 (34–50)	48 (34–59)		45 (34–54)	45 (34–54)	
**Age categories, years n (%)**						
5–11	34 (1.0)	143 (1)	Matching factor	1 (0.1)	5 (0.2)	Matching factor
12–17	65 (1.9)	241 (1.7)		8 (0.9)	33 (1.0)	
18–29	491 (14.1)	1964 (14.2)		120 (14.5)	483 (14.6)	
30–49	1256 (36.1)	4994 (36.0)		389 (47.2)	1554 (47.1)	
50–69	1094 (31.5)	4394 (31.7)		290 (35.2)	1151 (34.9)	
≥70	535 (15.4)	2139 (15.4)		17 (2.1)	71 (2.2)	
**Premedication use, n (%)**						
To prevent vaccine-related allergy (i.e., corticosteroids and antihistamines)	87 (2.5)	-	-	59 (7.2)	-	-
Painkillers/fever reducing drug	622 (17.9)	2138 (15.4)	<0.001	219 (26.5)	827 (25.1)	0.396
**Medical history (MedDRA PT), n (%)**						
Allergy	3476 (100)	-	-	825 (100)	-	-
Hay fever	2276 (65.5)	-	-	391 (47.4)	-	-
Allergy to medication	934 (26.9)	-	-	377 (45.7)	-	-
Dust mite allergy	1423 (40.9)	-	-	316 (38.3)	-	-
Allergy to animals	1117 (32.1)	-	-	202 (24.4)	-	-
Food allergy	775 (22.3)	-	-	185 (22.4)	-	-
Hypersensitivity	114 (3.3)	-	-	141 (17.1)	-	-
Allergy to insect bites	370 (10.6)	-	-	92 (11.1)	-	-
Other	293 (8.4)	-	-	85 (10.3)	-	-
Cardiovascular disorder	219 (6.3)	714 (5.1)	0.007	28 (3.4)	55 (1.7)	0.003
Diabetes Mellitus	103 (3.0)	410 (3)	1	12 (1.4)	55 (1.7)	0.759
Hypertension	443 (12.7)	1488 (10.7)	<0.001	83 (10.1)	227 (6.9)	0.003
Immunosuppression	100 (2.9)	235 (1.7)	<0.001	33 (4.0)	73 (2.2)	0.006
Liver disorder	16 (0.5)	29 (0.2)	0.015	6 (0.7)	9 (0.3)	0.095
Lung disorder	692 (19.9)	807 (5.8)	<0.001	159 (19.2)	119 (3.6)	<0.001
Mental disorder	261 (7.5)	555 (4)	<0.001	43 (5.2)	118 (3.6)	0.034
Malignant tumour	33 (0.9)	117 (0.8)	0.615	7 (0.8)	24 (0.7)	0.657
Nervous system disorder	47 (1.4)	161 (1.2)	0.399	10 (1.2)	20 (0.6)	0.104
Renal disorder	41 (1.2)	98 (0.7)	0.007	2 (0.2)	15 (0.5)	0.551
**Vaccine manufacturer, n (%)**						
Comirnaty	1494 (43.0)	5976 (43.1)	Matching factor	428 (51.9)	1712 (51.9)	Matching factor
Jcovden	344 (9.9)	1376 (9.9)		-	-	
Novavax	1 (<0.1)	1 (<0.1)		1 (0.1)	1 (<0.1)	
Spikevax	538 (15.5)	2139 (15.4)		391 (47.4)	1564 (47.4)	
Vaxzevria	1089 (31.3)	4356 (31.4)		3 (0.4)	12 (0.4)	
Unknown	10 (0.3)	29 (0.2)		2 (0.2)	8 (0.2)	

* Including 1 vaccinee aged 0–5 years; ** Including 2 vaccinees aged 0–5 years; ^a^ Vaccinees who reported receiving Novavax or an unknown COVID-19 vaccine will be excluded from the analysis due to the limited sample size. ^b^ Vaccinees who reported receiving Vaxzevria, Novavax or an unknown COVID-19 vaccine will be excluded from the analysis due to the limited sample size.

**Table 2:** Number of vaccinees with at least one follow-up questionnaire who received a first, second or booster dose of different COVID-19 vaccines and were included in the analyses.

	Comirnaty		Jcoven		Spikevax		Vaxzevria		Total	
	People with a History of Allergy	Matched Control	People with a History of Allergy	Matched Control	People with a History of Allergy	Matched Control	People with a History of Allergy	Matched Control	People with a History of Allergy	Matched Control
**Dose, n (%)**										
I	1494 (43.1)	5976 (43.2)	344 (9.9)	1376 (9.9)	538 (15.5)	2139 (15.4)	1089 (31.4)	4356 (31.5)	3465 (100)	13,847 (100)
II	1146 (51.3)	4584 (51.3)	-	-	405 (18.1)	1614 (18.1)	685 (30.6)	2740 (30.7)	2236 (100)	8938 (100)
Booster	428 (52.3)	1712 (52.3)	-	-	391 (47.7)	1564 (47.7)	-	-	819 (100)	3276 (100)

**Table 3:** Proportions of female and male participants who reported at least one serious ADR after receiving a first, second or booster dose of different COVID-19 vaccines, among people with a history of allergy and matched controls.

	At Least One Serious ADR											
	First Dose				Second Dose				Booster Dose			
	People with a History of Allergy		Matched Control		People with a History of Allergy		Matched Control		People with a History of Allergy		Matched Control	
	Female	Male	Female	Male	Female	Male	Female	Male	Female	Male	Female	Male
Number of participants, n (%)	2554 (100)	911 (100)	10,205 (100)	3642 (100)	1621 (100)	615 (100)	6478 (100)	2460 (100)	592 (100)	227 (100)	2368 (100)	908 (100)
**COVID-19 vaccines**												
Comirnaty, n (%)	1 (<0.1)	1 (0.1)	5 (<0.1)	2 (0.1)	4 (0.2)	2 (0.3)	3 (<0.1)	1 (<0.1)	1 (0.2)	0 (0)	2 (0.1)	2 (0.2)
Jcovden, n (%)	1 (<0.1)	0 (0)	1 (<0.1)	0 (0)	-	-	-	-	-	-	-	-
Spikevax, n (%)	1 (<0.1)	0 (0)	1 (<0.1)	1 (<0.1)	1 (0.1)	0 (0)	2 (<0.1)	0 (0)	1 (0.2)	0 (0)	1 (<0.1)	0 (0)
Vaxzevria, n (%)	2 (0.1)	0 (0)	5 (<0.1)	0 (0)	0 (0)	0 (0)	4 (0.1)	0 (0)	-	-	-	-
Total, n (%)	5 (0.2)	1 (0.1)	12 (0.1)	3 (0.1)	5 (0.3)	2 (0.3)	9 (0.1)	1 (<0.1)	2 (0.3)	0 (0)	3 (0.1)	2 (0.2)

**Table 4:** Allergic-related unsolicited ADRs most frequently reported by vaccinees after receiving a first dose of different COVID-19 vaccines, among people with a history of allergy and matched controls.

	First Dose											
	Comirnaty			Jcovden			Spikevax			Vaxzevria		
	People with a History of Allergy	Matched Controls	*p*-Value	People with a History of Allergy	Matched Controls	*p*-Value	People with a History of Allergy	Matched Controls	*p*-Value	People with a History of Allergy	Matched Controls	*p*-Value
Number of vaccinees, n (%)	1494	5976	Matching factor	344	1376	Matching factor	538	2139	Matching factor	1089	4356	Matching factor
Number of vaccinees reporting at least one unsolicited ADR, n (%)	360 (24.1)	1002 (16.8)	Matching factor	207 (60.2)	690 (50.1)	Matching factor	170 (31.6)	529 (24.7)	Matching factor	718 (65.9)	2554 (58.6)	Matching factor
**Allergic-related ADRs (PT), n (%)**												
Chest discomfort	0 (0)	9 (0.2)	0.219	2 (0.6)	3 (0.2)	0.262	1 (0.2)	1 (0)	0.361	6 (0.6)	20 (0.5)	0.628
Cough	11 (0.7)	11 (0.2)	0.001	2 (0.6)	11 (0.8)	1.000	2 (0.4)	3 (0.1)	0.264	10 (0.9)	26 (0.6)	0.292
Dyspnea	10 (0.7)	20 (0.3)	0.109	8 (2.3)	11 (0.8)	0.032	4 (0.7)	12 (0.6)	0.543	30 (2.8)	43 (1.0)	<0.001
Pruritus	8 (0.5)	11 (0.2)	0.033	2 (0.6)	2 (0.1)	0.180	7 (1.3)	4 (0.2)	0.002	9 (0.8)	17 (0.4)	0.081
Rash	4 (0.3)	11 (0.2)	0.518	4 (1.2)	3 (0.2)	0.033	4 (0.7)	1 (0)	0.006	9 (0.8)	11 (0.3)	0.009
Rash pruritic	5 (0.3)	8 (0.1)	0.153	1 (0.3)	0 (0)	0.200	3 (0.6)	4 (0.2)	0.149	7 (0.6)	5 (0.1)	0.003
Others *	48 (3.2)	34 (0.6)	<0.001	8 (2.3)	9 (0.7)	0.012	23 (4.3)	24 (1.1)	<0.001	33 (3)	33 (0.8)	<0.001

* Including one anaphylactic reaction and one anaphylactoid reaction following Jcovden and Comirnaty, respectively.

**Table 5:** Allergic-related unsolicited ADRs most frequently reported by vaccinees after receiving a second dose of different COVID-19 vaccines, among people with a history of allergy and matched controls.

	Second Dose								
	Comirnaty			Spikevax			Vaxzevria		
	People with a History of Allergy	Matched Controls	*p*-Value	People with a History of Allergy	Matched Controls	*p*-Value	People with a History of Allergy	Matched Controls	*p*-Value
Number of vaccinees, n (%)	1146	4584	Matching factor	405	1614	Matching factor	685	2740	Matching factor
Number of vaccinees reporting at least one unsolicited ADR, n (%)	293 (25.6)	807 (17.6)	Matching factor	217 (53.6)	803 (49.8)	Matching factor	145 (21.2)	407 (14.9)	Matching factor
**Allergic-related ADRs (PT), n (%)**									
Chest discomfort	5 (0.4)	6 (0.1)	0.051	1 (0.2)	2 (0.1)	0.489	0 (0)	1 (0)	1.000
Cough	2 (0.2)	2 (0)	0.180	3 (0.7)	8 (0.5)	0.469	2 (0.3)	6 (0.2)	0.664
Dyspnea	9 (0.8)	10 (0.2)	0.006	7 (1.7)	16 (1)	0.323	5 (0.7)	3 (0.1)	0.010
Pruritus	8 (0.7)	8 (0.2)	0.007	1 (0.2)	4 (0.2)	1.000	1 (0.1)	4 (0.1)	1.000
Rash	4 (0.3)	7 (0.2)	0.246	2 (0.5)	3 (0.2)	0.263	1 (0.1)	3 (0.1)	1.000
Rash pruritic	0 (0)	1 (0)	<0.001	1 (0.2)	1 (0.1)	0.361	0 (0)	0 (0)	-
Others *	23 (2)	29 (0.6)	<0.001	7 (1.7)	12 (0.7)	0.081	7 (1)	17 (0.6)	0.301

* Including one anaphylactoid reaction following Comirnaty.

**Table 6:** Allergic-related unsolicited ADRs most frequently reported by vaccinees after receiving a booster dose of different COVID-19 vaccines, among people with a history of allergy and matched controls.

	Booster Dose					
	Comirnaty			Spikevax		
	People with a History of Allergy	Matched Controls	*p*-Value	People with a History of Allergy	Matched Controls	*p*-Value
Number of vaccinees, n (%)	428 (100)	1712 (100)	Matching factor	391 (100)	1564 (100)	Matching factor
Number of vaccinees reporting at least one unsolicited ADR, n (%)	101 (23.6)	289 (16.9)	Matching factor	93 (23.8)	273 (17.5)	Matching factor
**Allergic-related ADRs (PT), n (%)**						
Chest discomfort	0 (0)	1 (0.1)	1.000	1 (0.3)	5 (0.3)	1.000
Cough	5 (1.2)	9 (0.5)	0.172	0 (0)	9 (0.6)	0.218
Dyspnea	4 (0.9)	12 (0.7)	0.541	4 (1)	6 (0.4)	0.120
Eczema	1 (0.2)	0 (0)	0.200	3 (0.8)	0 (0)	0.007
Pruritus	3 (0.7)	8 (0.5)	0.467	3 (0.8)	2 (0.1)	0.057
Rash	4 (0.9)	8 (0.5)	0.273	4 (1)	9 (0.6)	0.306

## Data Availability

Data are contained within the article and Supplementary Materials.

## Supplementary Materials

The following supporting information can be downloaded at: https://www.mdpi.com/article/10.3390/vaccines12091059/s1, Table S1: Participants recruited at the first vaccination cycle and booster dose, by Country; Table S2: Proportions of female and male participants who reported at least one ADR after receiving a first, second or booster dose of different COVID-19 vaccines, among people with a history of allergy and matched controls; Table S3: Proportions of female and male participants who reported at least one local solicited ADR after receiving a first, second or booster dose of different COVID-19 vaccines, among people with a history of allergy and matched controls; Table S4: Proportions of female and male participants who reported at least one systemic solicited ADR after receiving a first, second or booster dose of different COVID-19 vaccines, among people with a history of allergy and matched controls; Table S5: Frequency of reported local and systemic solicited ADRs following the first dose, second dose and booster dose of any vaccine, for people with a history of allergy and the matched control.
