# Supplementary material for: Safety of COVID-19 Vaccines among People with History of Allergy: A European Active Surveillance Study
Source: Vaccines (Basel). 2024 Sep 17;12(9):1059. doi: 10.3390/vaccines12091059 (PMC11435548; doi:10.3390/vaccines12091059)
Supplement: Supplementary file 1 [file vaccines-12-01059-s001.zip › supplementary material-2.pdf]

Supplementary table 1. Participants recruited at the first vaccination cycle and booster dose, by Country.

	First vaccination cycle		Booster dose	
	People with a history of allergy N= 3,476 (%)	Matched control N= 13,877 (%)	People with a history of allergy N= 825 (%)	Matched control N= 3,297 (%)
Belgium	7 (0.2)	21 (0.2)	-	-
France	192 (5.5)	682 (4.9)	463 (56.1)	2,329 (70.6)
Ireland	-	-	11 (1.3)	72 (2.2)
Italy	186 (5.4)	512 (3.7)	235 (28.5)	436 (13.1)
Netherlands	3,045 (87.6)	12,416 (89.5)	-	-
Portugal	-	3 (<0.1)	15 (1.8)	9 (0.3)
Romania	5 (0.1)	35 (0.3)	19 (2.3)	19 (0.6)
Slovakia	12 (0.3)	28 (0.2)	2 (0.2)	3 (0.1)
Spain	2 (0.1)	10 (0.1)	31 (3.8)	149 (4.5)
Switzerland	1 (<0.1)	8 (<0.1)	11 (1.3)	82 (2.5)
UK	26 (0.7)	162 (1.2)	38 (4.6)	198 (6.0)

Supplementary table 2. Proportions of female and male participants who reported at least one ADR after receiving a first, second or booster dose of different COVID-19 vaccines, among people with a history of allergy and matched controls.

	At least one ADR											
	First dose				Second dose				Booster dose			
	People with a history of allergy		Matched control		People with a history of allergy		Matched control		People with a history of allergy		Matched control	
	female	male	female	male	female	male	female	male	female	male	female	male
Number of participants, n (%)	2,554 (100)	911 (100)	10,205 (100)	3,642 (100)	1,621 (100)	615 (100)	6,478 (100)	2,460 (100)	592 (100)	227 (100)	2,368 (100)	908 (100)
**COVID-19 vaccines**												
Comirnaty, n (%)	774 (30.3)	367 (40.3)	2,782 (27.3)	1,182 (32.5)	602 (37.1)	303 (49.3)	2,139 (33.0)	943 (38.3)	232 (39.2)	72 (31.7)	788 (33.3)	264 (29.1)
Jcoven, n (%)	239 (9.4)	70 (7.7)	883 (8.7)	251 (6.9)	-	-	-	-	-	-	-	-
Spikevax, n (%)	372 (14.6)	134 (14.7)	1,457 (14.3)	520 (14.3)	284 (17.5)	104 (16.9)	1,126 (17.4)	414 (16.8)	238 (40.2)	78 (34.4)	827 (34.9)	244 (26.9)
Vaxzevria, n (%)	937 (36.7)	107 (11.7)	3,688 (36.1)	395 (10.8)	596 (36.8)	59 (9.6)	2,333 (36.0)	216 (8.8)	-	-	-	-
Total, n (%)	2,322 (90.9)	678 (74.4)	8,810 (86.3)	2,348 (64.5)	1,482 (91.4)	466 (75.8)	5,598 (86.4)	1,573 (63.9)	470 (79.4)	150 (66.1)	1,615 (68.2)	508 (55.9)

Supplementary table 3. Proportions of female and male participants who reported at least one local solicited ADR after receiving a first, second or booster dose of different COVID-19 vaccines, among people with a history of allergy and matched controls.

	At least one local solicited ADR											
	First dose				Second dose				Booster dose			
	People with a history of allergy		Matched control		People with a history of allergy		Matched control		People with a history of allergy		Matched control	
	female	male	female	male	female	male	female	male	female	male	female	male
Number of participants, n (%)	2,554 (100)	911 (100)	10,205 (100)	3,642 (100)	1,621 (100)	615 (100)	6,478 (100)	2,460 (100)	592 (100)	227 (100)	2,368 (100)	908 (100)
**COVID-19 vaccines**												
Comirnaty, n (%)	429 (16.8)	164 (18.0)	1,432 (14.0)	515 (14.1)	262 (16.2)	106 (17.2)	829 (12.8)	304 (12.4)	170 (28.7)	44 (19.4)	507 (21.4)	150 (16.5)
Jcovden, n (%)	121 (4.7)	38 (4.2)	442 (4.3)	84 (2.3)	-	-	-	-	-	-	-	-
Spikevax, n (%)	249 (9.7)	66 (7.2)	913 (8.9)	260 (7.1)	168 (10.4)	50 (8.1)	694 (10.7)	200 (8.1)	175 (29.6)	57 (25.1)	589 (24.9)	144 (15.6)
Vaxzevria, n (%)	591 (23.1)	48 (5.3)	2,214 (21.7)	219 (6.0)	169 (10.4)	11 (1.8)	645 (10.0)	38 (1.5)	-	-	-	-
Total, n (%)	1,390 (54.4)	316 (34.7)	5,001 (49.0)	1,078 (29.6)	599 (37.0)	167 (27.2)	2,168 (33.5)	542 (22.0)	345 (58.3)	101 (44.5)	1,096 (46.3)	294 (32.4)

Supplementary table 4. Proportions of female and male participants who reported at least one systemic solicited ADR after receiving a first, second or booster dose of different COVID-19 vaccines, among people with a history of allergy and matched controls.

	At least one systemic solicited ADR											
	First dose				Second dose				Booster dose			
	People with a history of allergy		Matched control		People with a history of allergy		Matched control		People with a history of allergy		Matched control	
	female	male	female	male	female	male	female	male	female	male	female	male
Number of participants, n (%)	2,554 (100)	911 (100)	10,205 (100)	3,642 (100)	1,621 (100)	615 (100)	6,478 (100)	2,460 (100)	592 (100)	227 (100)	2,368 (100)	908 (100)
**COVID-19 vaccines**												
Comirnaty, n (%)	527 (20.6)	201 (22.1)	1,764 (17.3)	639 (17.5)	396 (24.4)	170 (27.6)	1,263 (19.5)	515 (20.9)	203 (34.3)	61 (26.9)	638 (26.9)	198 (21.8)
Jcovden, n (%)	220 (8.6)	88 (9.7)	826 (8.1)	240 (6.6)	-	-	-	-	-	-	-	-
Spikevax, n (%)	263 (10.3)	88 (9.7)	902 (8.8)	310 (8.5)	239 (14.7)	91 (14.8)	972 (15.0)	352 (14.3)	211 (35.6)	68 (30.0)	713 (30.1)	210 (23.1)
Vaxzevria, n (%)	874 (34.2)	99 (10.9)	3,397 (33.3)	365 (10.0)	293 (18.1)	22 (3.6)	1,052 (16.2)	68 (2.8)	-	-	-	-
Total, n (%)	1,884 (73.8)	476 (52.3)	6,889 (67.5)	1,554 (42.7)	928 (57.2)	283 (46.0)	3,287 (50.7)	935 (38.0)	414 (69.9)	129 (56.8)	1,351 (57.1)	408 (44.9)

Supplementary table 5. Frequency of reported local and systemic solicited ADRs following the first dose, second dose and booster dose of any vaccine, for people with a history of allergy and the matched control.

	**First dose**			**Second dose**			**Booster dose**		
	**People with a history of allergy N= 3,476 (%)**	**Matched control N= 13,877 (%)**	*p value*	**People with a history of allergy N= 2,240 (%)**	**Matched control N= 8,951 (%)**	*p value*	**People with a history of allergy N= 825 (%)**	**Matched control N= 3,297 (%)**	*p value*
**Local solicited ADR, (MedDRA PT), n (%)**									
Injection site erythema	241 (6.9)	727 (5.2)	<0.001	137 (6.1)	475 (5.3)	0.145	62 (7.5)	169 (5.1)	0.009
Injection site haematoma	151 (4.3)	525 (3.8)	0.139	70 (3.1)	243 (2.7)	0.326	24 (2.9)	70 (2.1)	0.221
Injection site induration	40 (1.2)	133 (1)	0.354	7 (0.3)	18 (0.2)	0.454	5 (0.6)	14 (0.4)	0.562
Injection site inflammation	594 (17.1)	1,874 (13.5)	<0.001	266 (11.9)	894 (10)	0.009	156 (18.9)	458 (13.9)	<0.001
Injection site pain	1,509 (43.4)	5,346 (38.5)	<0.001	648 (28.9)	2,313 (25.8)	0.003	410 (49.7)	1,259 (38.2)	<0.001
Injection site pruritus	127 (3.7)	368 (2.7)	0.002	56 (2.5)	189 (2.1)	0.296	41 (5)	100 (3)	0.008
Injection site reaction	3 (0.1)	9 (0.1)	0.716	3 (0.1)	7 (0.1)	0.429	2 (0.2)	9 (0.3)	1.000
Injection site swelling	511 (14.7)	1,671 (12)	<0.001	207 (9.2)	727 (8.1)	0.094	166 (20.1)	461 (14)	<0.001
Injection site warmth	374 (10.8)	1,123 (8.1)	<0.001	201 (9)	631 (7)	0.002	71 (8.6)	190 (5.8)	0.003
**Systemic solicited ADR, (MedDRA PT), n (%)**									
Arthralgia	596 (17.1)	1,944 (14.0)	<0.001	236 (10.5)	673 (7.5)	<0.001	142 (17.2)	410 (12.4)	<0.001
Chills	927 (26.7)	3,484 (25.1)	0.061	296 (13.2)	1,111 (12.4)	0.322	200 (24.2)	608 (18.4)	<0.001
Fatigue	1,502 (43.2)	5,266 (37.9)	<0.001	720 (32.1)	2,479 (27.7)	<0.001	392 (47.5)	1,159 (35.2)	<0.001
Headache	1,315 (37.8)	4,788 (34.5)	<0.001	588 (26.3)	1,953 (21.8)	<0.001	293 (35.5)	869 (26.4)	<0.001
Malaise	1,302 (37.5)	4,514 (32.5)	<0.001	621 (27.7)	2,053 (22.9)	<0.001	257 (31.2)	734 (22.3)	<0.001
Myalgia	1,373 (39.5)	4,885 (35.2)	<0.001	596 (26.6)	1,999 (22.3)	<0.001	276 (33.5)	864 (26.2)	<0.001
Nausea	613 (17.6)	1954 (14.1)	<0.001	252 (11.3)	738 (8.2)	<0.001	117 (14.2)	258 (7.8)	<0.001
Body temperature increased	121 (3.5)	462 (3.3)	0.695	79 (3.5)	254 (2.8)	0.099	70 (8.5)	172 (5.2)	<0.001
Pyrexia	702 (20.2)	2,525 (18.2)	0.007	262 (11.7)	874 (9.8)	0.007	140 (17)	405 (12.3)	<0.001
Hyperpyrexia	13 (0.4)	49 (0.4)	0.979	4 (0.2)	5 (0.1)	0.085	1 (0.1)	4 (0.1)	1.000
